# Primary squamous cell carcinoma of thyroid: a case report and review of literature

**DOI:** 10.1186/1758-3284-4-8

**Published:** 2012-03-27

**Authors:** Mutahir A Tunio, Mushabbab Al Asiri, Mosa Fagih, Rashad Akasha

**Affiliations:** 1 Department of Radiation Oncology, Comprehensive Cancer Center, King Fahad Medical City, Riyadh, PO 59046, Saudi Arabia; 2 Department of Cytogenetics, King Fahad Medical City, Riyadh, PO 59046, Saudi Arabia; 3 Resident Radiation Oncology, Department of Radiation Oncology, Comprehensive Cancer Center, King Fahad Medical City, Riyadh, PO 59046, Saudi Arabia

**Keywords:** Squamous cell carcinoma, Thyroid, Rare, Primary, Fatal

## Abstract

**Background:**

Thyroid gland lacks squamous epithelium (except in some rare situations like embroyonic remnants or in inflammatory processes); for that reason the primary squamous cell carcinoma (SCC) of thyroid is extremely rare entity, seen only in less than 1% of all thyroid malignancies and is considered almost fatal. So, far, only few case reports have been published in literature.

**Case presentation:**

Herein we present a 54 years old Saudi female with 3 months history of progressive neck swelling and hoarse voice, who was referred to us by her primary care physician as suspected case of anaplastic carcinoma of thyroid for radical external beam radiation therapy (EBRT). Fine Needle aspiration cytology (FNAC) revealed squamous cell carcinoma. Computed tomography (CT) neck showed 10 × 10 cm mass in left lobe of thyroid invading trachea and skin. Extensive staging work up ruled out the possibility of any primary site of SCC other than thyroid gland. Tumor was found unresectable and was referred to radiation oncology. She received palliative EBRT 30 Gy in 10 fractions. After completion of EBRT, there was progression of disease and patient died 3 months after completion of EBRT by airway compromise.

**Conclusion:**

Primary SCC of thyroid is rare and aggressive entity. FNAC is reliable and effective tool for immediate diagnosis. Surgery is a curative option, but it is not always possible as most of cases present as locally advanced with adjacent organs involvement. EBRT alone was found ineffective. Aggressive combined modality (debulking surgery, radiation and chemotherapy) shall be considered for such cases.

## Background

Primary squamous cell carcinoma (SCC) of thyroid is an uncommon malignancy and has poor prognosis [1]. SCC of thyroid constitutes less than 1% of thyroid malignancies and has been found fatal within one year of initial diagnosis [2]. The median age is fifth and sixth decade, but can be seen at any age. Main cause of death in these patients is secondary to respiratory interference by direct invasion or compression of the trachea [3]. When SCC of thyroid is diagnosed, the possibility of the tumor arising from adjacent organs (esophagus, larynx) or representing metastatic disease from primary growth somewhere else (lungs) must be considered before concluding the malignancy as SCC of thyroid.

The etiology of SCC thyroid is uncertain as thyroid gland lacks the squamous epithelium. However three theories have been postulated; first the *embryonic nest theory *suggests that squamous cells are derived from the embryonic remnants such thyroglossal duct, thymic epithelium and ultimobronchial body [4]. Second the *metaplasia theory *suggests that the environmental stimuli (inflammation and Hashimoto's thyroiditis) result in squamous metaplasia [5]. Third the *de-differentiation theory *suggests that existing papillary, follicular, medullary and anaplastic thyroid carcinoma de-differentiate into SCC [6,7].

Herein we present a case of 54 years old Saudi lady with locally advanced primary squamous cell carcinoma of thyroid, diagnosed by fine needle aspiration cytology (FNAC) was treated with radiation therapy.

## Case presentation

A 54 year old Saudi female presented in our clinic with neck swelling and hoarse voice. She had noticed this swelling for 3 months and it had been rapidly increasing in size over a week causing dyspnoea and dysphagia to solids. Her previous medical history revealed type II diabetes mellitus since last 10 years and hypothyroidism since last 3 years, for that she was taking thyroxin 50 micrograms daily and metformin. She had no history of smoking and her weight was stable.

**Figure 1:**
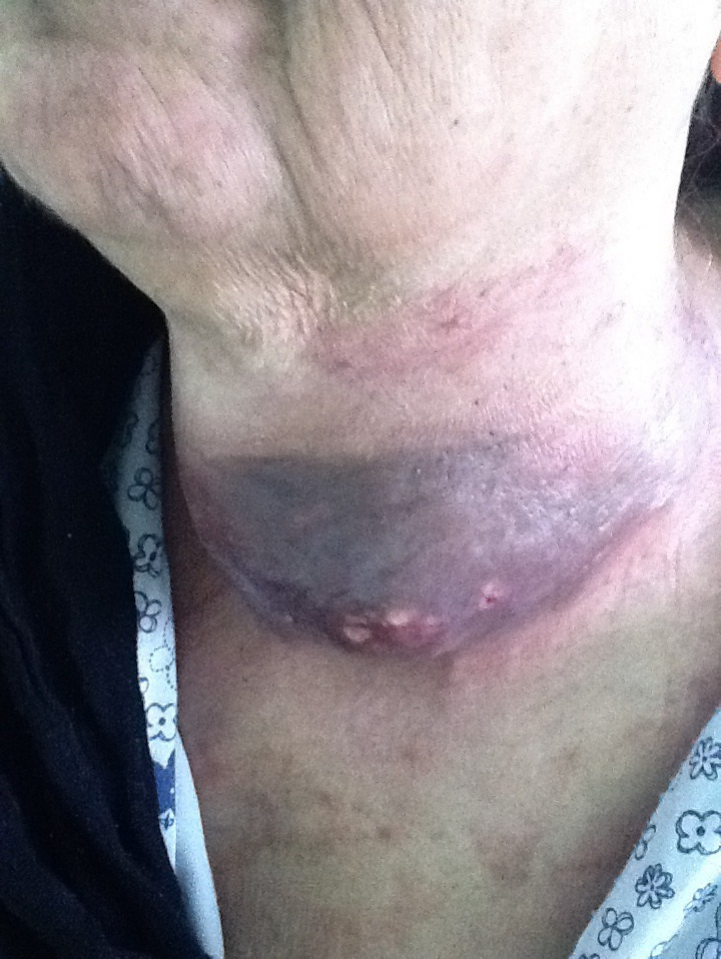
**A fixed hard neck mass of size 8 × 8 cm was palpable in the left thyroid lobe with inflammatory surface**.

On physical examination, her vitals were stable. A fixed hard neck mass of size 8 × 8 cm was palpable in the left thyroid lobe with inflammatory surface Figure 1. There was no palpable cervical lymphadenopathy and examination of chest, heart, nervous system and abdomen was normal. Clinical differential diagnosis was anaplastic carcinoma of thyroid.

**Figure 2:**
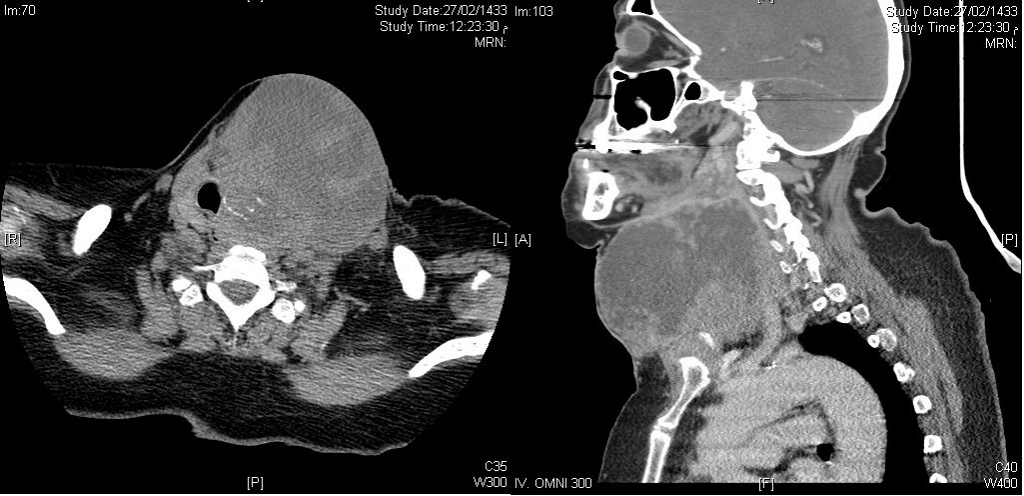
**Computed tomography (CT) neck showing 10 × 10 cm mass in left lobe of thyroid, partially necrotic invading to adjacent skin and trachea and no cervical lymphadenopathy**.

**Figure 3:**
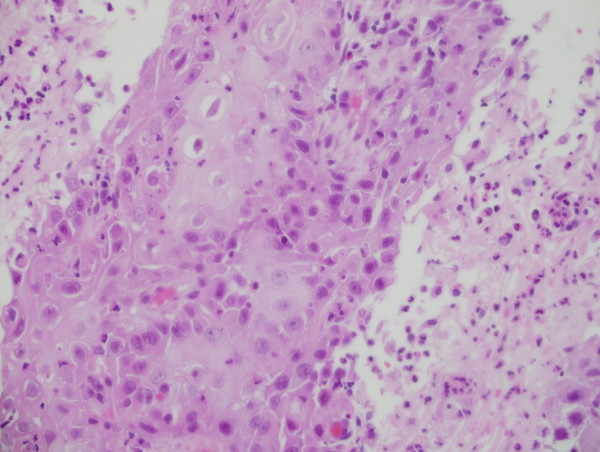
**Fine needle aspiration cytology (FNAC) showing nests of pleomorphic cells with abundant eosinophilic cytoplasm and keratin formation along with intercellular bridging**.

**Figure 4:**
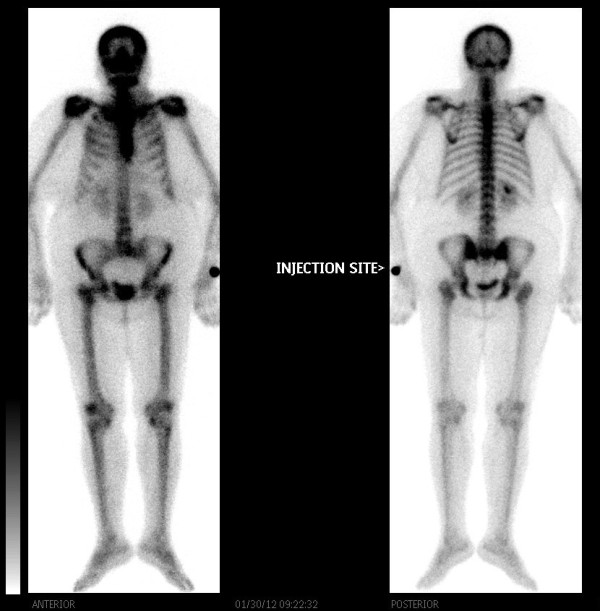
**Bone scintigraphy showing no evidence of distant bone metastasis**.

Ultrasonography showed huge left thyroid lobe partially cystic and solid mass of size 8.5 × 9 cm. Computed tomography (CT) neck showed 10 × 10 cm mass in left lobe of thyroid, partially necrotic invading to adjacent skin and trachea and no lymphadenopathy was found Figure 2. Serum T4, thyroid stimulating hormone (TSH), thyroglobulin and serum calcium were within normal limits. Fine needle aspiration cytology (FNAC) of mass was performed, which revealed squamous cell carcinoma Figure 3. Differential diagnosis was metastatic squamous cell carcinoma from another primary location. CT chest, abdomen, pelvis, magnetic resonance imaging (MRI) of head and neck region, pan-endoscopy, laryngoscopy, esophagoscopy and bone scintigraphy did not reveal any primary lesion or other metastatic disease Figure 4. Radiological stage was made as T4N0M0.

In a multidisciplinary tumor (MDT) meeting it was labeled unresectable and patient was referred for external beam radiation therapy (EBRT) after prophylactic percutaneous endoscopic gastrostomy (PEG) insertion. Due to retrosternal extention of disease, tracheostomy was deferred. Patient received 30 Grays (Gy) in 10 fractions to thyroid. Post radiation therapy, there was progression size of neck mass with progressive dyspnoea. Patient died of airway compromise 3 months of palliative EBRT.

## Discussion

Primary SCC of the thyroid gland is an extremely rare and aggressive entity usually presents with classic triad features; (I) rapidly enlarging mass in the older patients behaving like anaplastic carcinoma, (II) it may be associated with other thyroid malignancies and (III) histological features of intercellular bridges and keratin [8-10]. FNAC is reliable and confirmatory tool, but it is mandatory to exclude the metastatic SCC [11].

Treatment with surgery, radiation therapy and chemotherapy alone has been found ineffective in previously published similar case reports, as majority of these patients present as locally advanced cases not amenable for curative resection Table 1. The better survival rates have been achieved with aggressive combination therapy (surgery followed by adjuvant radiation therapy (50-60 Gy) with or without chemotherapy or induction chemotherapy followed by surgery) [11-16].

**Table 1:** Previously published case reports (2000-2012) of primary squamous cell carcinoma of thyroid

Author [Ref]	Gender/Age	Presentation	Stage	Associated Problem	Treatment given	Survival	Comments
Zimmer PW [1]	Female/64 years	Asymptomatic neck mass	T2N0M0	-	Total thyroidectomy	7 months	-

Kebapci N [7]	Female/25 years	Right neck mass	T4N1M0	Hashimotos' thyroiditis			

Papillary carcinoma	Total thyroidectomy and RAI therapy	44 months	-				

Ko YS [8]	Male/87 years	Asymptomatic neck mass	T4N0M0	-	Right lobectomy	NA	CK5/6 + CK19 + EMA,p53 focal + BRAF mutation +

Mercante G [9]	Male/67 years	-	T2N0M0	Follicular carcinoma	Lobe-isthmusectomy + Adjuvant chemoradiation	2 years	-

De Vos FY [10]	-	Neck mass	T4N0M0	-	Induction chemotherapy (Cisplatin + paclitaxel)		

Total thyroidectomy	20 months	Induction chemotherapy resulted in R0 resection					

Yucel H [11]	Male/88 years	Neck mass	T4N0M0	Hyperthyroidism	Total thyroidectomy Adjuvant radiation therapy	6 months	Patient RAI therapy 20 years back

Eorn TI [12]	Female/43 years	Neck mass	T3N0M0	Papillary carcinoma	Total thyroidectomy Adjuvant radiation therapy 59.4 Gy And RAI	8 months	CK7 + p 63 +

Makay O [13]	Male/53 years Male/71 years	Neck mass, hoarse voice and weight loss	T3N0M0	-	Near total thyroidectomy Chemoradiation 50 Gy + Doxorubicin and cyclophosphamide	2 months 4 months 5 months	-

Fassan M [17]	Female/64 years	Neck mass	T3N0M0	Goiter	Total thyroidectomy	NA	CK 5/6 + CK 7 + CK 19 +

Maamouri F [18]	Female/87 years	Right neck mass	T3N0M0	Papillary carcinoma	Total thyroidectomy And RAI therapy	6 months	-

Chintamani [14]	Female/50 years Male/60 years Male/58 years	Dysphagia, hoarse voice and stridor	T4N0M0	Hyperthyroidism	Total thyroidectomy Adjuvant radiotherapy 50 Gy	12 months	-

Jung TS [15]	Male/56 years	Neck mass, hoarse voice	T3N0M0	Follicular carcinoma	Total thyroidectomy Adjuvant radiotherapy 50 Gy	8 years	-

Sutak J [19]	Female/80 years	Asymptomatic neck mass	T4N1M0	Tall cell variant papillary carcinoma	Total thyroidectomy	-	CK 7 + CK 19 + CK AE1/3 + P53 focal +

Zhou XH [16]	4 patients	NA	T4N0M0	-	Total thyroidectomy Adjuvant radiotherapy 50 Gy + chemotherapy	4 months 6 months 13 months 26 months	Longer survival was seen in combined trimodality treatment

Lam KY [20]	4 females/71 years	Neck mass, stridor	T4N0M0	-	Total thyroidectomy	4 months	CK 7 + CK 19 + CK AE1/3 + P53 focal +

Kleer CG [21]	7 females/1 male 31-90 years	Neck mass	T4N0M0	Tall cell variant papillary carcinoma	Total thyroidectomy	6 Months- 48 months	-

Jones JM [22]	Male/48 years	Hoarse voice, left neck mass	T4N1M0	-	Total thyroidectomy and LND	8 months	-

## Conclusion

Primary squamous cell carcinoma of thyroid is a rare and aggressive entity with poor prognosis. FNAC is effective confirmatory tool, but efforts shall be made to rule out metastatic SCC originating from other sites. Surgery, radiotherapy and chemotherapy alone are ineffective. Aggressive treatment with surgery followed by adjuvant radiotherapy with or without chemotherapy is recommended to achieve better outcome.

## Consent

Written permission was taken from the patient for publication of the case report.

## Abbreviations

SCC: Squamous cell carcinoma; EBRT: External beam radiation therapy; FNAC: Fine needle aspiration cytology; CT: Computed tomography; RAI: Radioactive iodine; TSH: Thyroid stimulating hormone; MDT: Multidisciplinary tumor meeting; PEG: Percutaneous endoscopic gastrostomy.

## Competing interests

Authors have neither potential conflict of interest nor received any grants for this case report.

## Authors' contributions

MAT, MAA Manuscript preparation. RA Data Collection. MF Pathological data. All authors read and approved the final manuscript.

## References

[B1] Zimmer PW Wilson D Bell N Primary squamous cell carcinoma of the thyroid gland Mil Med 2003 168 124 5 12636140

[B2] Korovin GS Cho HT Kuriloff DB Sobol SM Squamous cell carcinoma of the thyroid: a diagnostic dilemma Ann Otol Rhinol Laryngol 1989 98 59 65 2910191 10.1177/000348948909800113

[B3] Simpson WJ Carruthers TH Squamous cell carcinoma of thyroid gland Am J Surg 1988 156 44 6 10.1016/S0002-9610(88)80169-7 3394892

[B4] Goldberg HM Harrey P Squamous cell cysts of the thyroid with special reference to the etiology of squamous epithelium in the human thyroid Br J Surg 1956 43 565 9 10.1002/bjs.18004318203 13342413

[B5] Chaudhary RK Barnes EL Myers EN Squamous cell carcinoma arising in Hashimoto's thyroiditis Head Neck 1994 16 582 5 10.1002/hed.2880160615 7822183

[B6] Bronner MP LiVolsi VA Spindle cell squamous carcinoma of the thyroid: an unusual anaplastic tumor associated with tall cell papillary carcinoma Mod Pathol 1991 4 630 43 1722043

[B7] Kebapci N Efe B Kabukcuoglu S Akalin A Kebapci M Diffuse sclerosing variant of papillary thyroid carcinoma with papillary squamous cell carcinoma J Endocr Invest 2002 25 730 4 12240907 10.1007/BF03345109

[B8] Ko YS Hwang TS Han HS Lim SD Kim WS Oh SY Primary pure squamous cell carcinoma of the thyroid: report and histogenic consideration of a case involving a BRAF mutation Pathol Int 2012 62 43 8 10.1111/j.1440-1827.2011.02745.x 22192803

[B9] Mercante G Marchesi A Covello R Dainese L Spiano G Mixed squamous cell carcinoma and follicular carcinoma of the thyroid gland Auris Nasus Larynx 2011 8 1 5 PMID 21855238 10.1016/j.anl.2011.07.003 21855238

[B10] De Vos FY Sewnaik A de Witt JH Smid EJ den Bakker MA van Meerten E Combined therapy for thyroid squamous cell carcinoma Head Neck 2012 34 131 4 10.1002/hed.21484 20652981

[B11] Yucel H Schaper NC van Beek M Bravenboer B Primary squamous cell carcinoma of the thyroid years after radioactive iodine treatment Neth J Med 2010 68 224 6 20508272

[B12] Eorn TI Koo BY Kim BS Kang KH Jung SK Jun SY Bae HS Kim LS Coexistence of primary squamous cell carcinoma of thyroid with classic papillary thyroid carcinoma Pathol Int 2008 58 797 800 10.1111/j.1440-1827.2008.02314.x 19067856

[B13] Makay O Kaya T Ertan T Icoz G Akyildiz M Yilmaz M Tuncyurek M Yetkin E Primary squamous cell carcinoma of the thyroid: report of three cases Endocr J 2008 55 359 64 10.1507/endocrj.K07E-102 18379125

[B14] Chintamani PK Singh J Sugandhi N Bansal A Bhatnagar D Saxena S Is an aggressive approach justified in the management of an aggressive cancer- the squamous cell carcinoma of thyroid? Int Semin Surg Oncol 2007 4 8 10.1186/1477-7800-4-8 17397523 PMC1847680

[B15] Jung TS Oh YL Min YK Lee MS Lee MK Kim KW Chung JH Korean J Intern Med 2006 21 73 8 10.3904/kjim.2006.21.1.73 16646570 PMC3891069

[B16] Zhou XH Primary squamous cell carcinoma of the thyroid Eur J Surg Oncol 2002 28 42 5 10.1053/ejso.2001.1180 11869012

[B17] Fassan M Pennelli G Pelizzo MR Rugge M Primary squamous cell carcinoma of the thyroid. Immunohistochemical profile and literature review Tumori 2007 93 518 21 18038891 10.1177/030089160709300522

[B18] Maamouri F Goucha A Ben Mna N Ben Hassouna J Debbabi B Ouslati Z Boussen H El May A Gamoudi A Tunis Med 2007 85 251 3 17668586

[B19] Sutak J Armstrong JS Rusby JE Squamous cell carcinoma arising in a tall cell papillary carcinoma of the thyroid J Clin Pathol 2005 58 662 4 10.1136/jcp.2004.021451 15917423 PMC1770674

[B20] Lam KY Lo CY Liu MC Primary squamous cell carcinoma of thyroid gland: an entity with aggressive clinical behavior and distinctive cytokeratin expression profiles Histopathology 2001 39 279 86 10.1046/j.1365-2559.2001.01207.x 11532039

[B21] Kleer CG Giordano TJ Merino MJ Squamous cell carcinoma of the thyroid: an aggressive tumor associated with tall cell variant of papillary thyroid carcinoma Mod Pathol 2000 13 742 6 10.1038/modpathol.3880129 10912933

[B22] Jones JM McCluggage WG Russell CF Primary squamous cell carcinoma of the thyroid Ulster Med J 2000 69 58 60 10881647 PMC2449177

